# Accuracy of noninvasive transcutaneous carbon dioxide monitoring in preterm neonates and very low birth weight infants compared with larger neonates

**DOI:** 10.3389/fped.2026.1794358

**Published:** 2026-03-31

**Authors:** Chia-Ling Li, Yi-Li Hung, Chung-Min Shen, Wu-Shiun Hsieh

**Affiliations:** 1 Departments of Pediatrics, Cathay General Hospital, Taipei, Taiwan; 2 Departments of Pediatrics, Dianthus Maternal Fetal Medicine Clinic, Taipei, Taiwan; 3 School of Medicine, Fu Jen Catholic University, New Taipei City, Taiwan; 4 School of Medicine, National Tsing Hua University, Hsinchu, Taiwan; 5 Department of Pediatrics, National Taiwan University Children’s Hospital, Taipei, Taiwan; 6 Department of Pediatrics, National Taiwan University College of Medicine, Taipei, Taiwan; 7 School of Medicine, College of Medicine, Kaohsiung Medical University, Kaohsiung, Taiwan

**Keywords:** gestational age, premature, transcutaneous carbon dioxide, very low birth weight, very preterm

## Abstract

**Purpose:**

Carbon dioxide monitoring is particularly critical in very preterm and very low birth weight (VLBW) infants during the first weeks of life because of the hypercarbia and hypocarbia related morbidities. Conventional methods for measuring partial pressure of carbon dioxide in arterial blood (PaCO_2_) are challenging for preterm neonates due to complications. Continuous transcutaneous partial pressure of carbon dioxide (TcPCO_2_) measurement is a well-established method in neonatal intensive care unit. This study primarily aimed to evaluate the accuracy and reliability of transcutaneous partial pressure of CO_2_ (TcPCO_2_) compared with PaCO_2_ in preterm neonates and VLBW infants (birth weight <1,500 g), and secondarily to explore how accuracy varies across different gestational age and birth weight strata.

**Methods:**

This retrospective single-center observational study included a heterogeneous neonatal intensive care unit (NICU) cohort. In neonates admitted to the NICU for respiratory distress, PaCO_2_ and TcPCO_2_ were measured simultaneously. Statistical analysis included linear regression, calculation of intraclass correlation coefficients (ICC), repeated measures correlation (rmcorr), and the repeated-measures Bland-Altman analysis.

**Results:**

A total of 143 infants (mean gestational age: 35 ± 3.2 weeks; mean birth weight: 2,374 ± 694.4 g) were recruited, and 410 PaCO_2_ and TcPCO_2_ measurement pairs were obtained for analysis. A strong correlation (rmcorr = 0.800) with an intraclass correlation coefficient (ICC) of 0.93 (95% CI: 0.91–0.94, *p* < 0.001) was found in preterm neonates, particularly those with gestational age below 32 weeks, or a birth weight below 1,500 g. Similar correlations were observed in larger infants.

**Conclusions:**

TcPCO_2_ is a noninvasive and reliable method for continuous respiratory monitoring in preterm neonates and very low birth weight infants during the early postnatal period. TcPCO_2_ is useful for continuous trend monitoring and may reduce the need for arterial sampling; however, confirmatory blood gas testing is recommended for extreme values or unstable clinical conditions.

## Introduction

Very preterm and very low birth weight (VLBW) infants are particularly vulnerable to both hypo- and hypercapnia in the first weeks of life, when cerebral and pulmonary circulation are highly unstable (1–3). Measuring the partial pressure of CO_2_ in arterial blood (PaCO_2_) is the gold standard for assessing gas exchange. However, routine measurement of PaCO_2_ in preterm neonates requires insertion of an arterial catheter, which can lead to severe complications, such as arterial spasm, thrombosis, ischemia, and iatrogenic anemia (4, 5). In addition, arterial blood gas sampling cannot provide continuous monitoring. Reliable noninvasive CO_2_ monitoring is therefore especially important in this high-risk population.

Noninvasive methods for continuous CO_2_ monitoring include end-tidal CO_2_ and transcutaneous partial pressure of CO_2_ (TcPCO_2_) measurement (6). However, end-tidal CO_2_ monitoring is unreliable in infants with severe lung disease and ventilation perfusion mismatch and cannot be used in infants without an endotracheal tube (6). Current respiratory care guidelines recommend permissive hypercapnia with lower ventilator settings to prevent barotrauma and bronchopulmonary dysplasia in preterm neonates (7).

TcPCO_2_ measurement may facilitate ventilator titration and permissive hypercapnia, potentially reducing ventilator-induced lung injury (8). It could provide a noninvasive and accurate method for continuously monitoring the respiration status in neonates. Although previous studies have evaluated TcPCO_2_ accuracy in mixed neonatal cohorts, few have focused specifically on very preterm or VLBW infants and on how accuracy and agreement evolve across gestational age and birth weight groups (8–11). Therefore, the primary aim of this study was to assess the accuracy and reliability of TcPCO_2_ in preterm neonates and VLBW infants (birth weight <1,500 g) during the early postnatal period. A secondary aim was to explore how the agreement between TcPCO_2_ and PaCO_2_ varies across gestational age and birth weight strata in a heterogeneous NICU population.

## Materials and methods

This study was approved by the Institutional Review Board of Cathay General Hospital (CGH-P112015) on June 5, 2023.

### Study design and primary population

**Figure 1:**
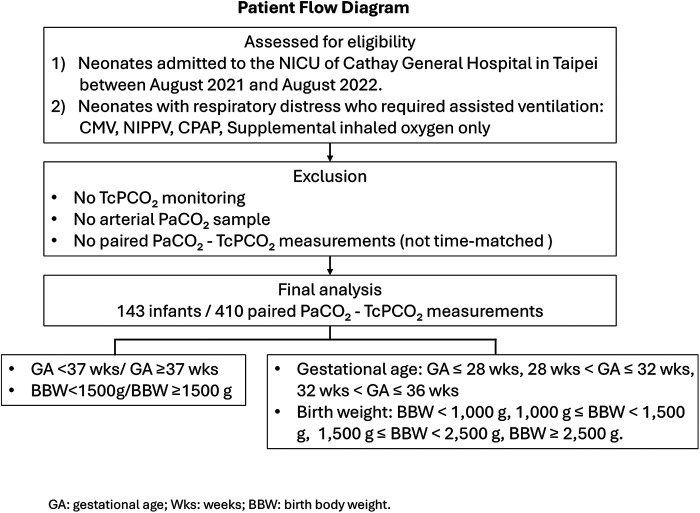
Patient flow diagram.

A retrospective chart review was conducted of neonates admitted to the NICU of Cathay General Hospital in Taipei between August 2021 and August 2022. This study included neonates with respiratory distress who required assisted ventilation, including continuous mandatory ventilation, noninvasive positive pressure ventilation, continuous positive airway pressure (CPAP), or supplemental inhaled oxygen only (Supplementary Table S1). For the primary analysis, we focused on preterm neonates (gestational age <37 weeks) and/or VLBW infants (birth weight <1,500 g), in whom TcPCO_2_ monitoring is most clinically relevant during the early postnatal period. Infants with higher gestational age or birth weight were included in secondary analyses to explore how TcPCO_2_ performance varies across maturational strata Figure 1.

For infants admitted due to respiratory distress, TcPCO_2_ monitoring was initiated on the first day of hospitalization. In more severe cases requiring intubation and mechanical ventilation, arterial blood gases were typically obtained according to clinical condition, while TcPCO_2_ was monitored continuously with scheduled site recalibration every 2 h. For infants receiving only supplemental oxygen or CPAP, measurements were performed once daily until respiratory support or oxygen therapy was discontinued. Therefore, multiple paired data points per patient per day could be included in the analysis.

Whenever PaCO_2_ from arterial blood was measured, the corresponding TcPCO_2_ values were recorded simultaneously. Transcutaneous CO_2_ was measured using the SenTec (SenTec AG, Therwil, Switzerland) transcutaneous monitor with an OxiVenT sensor. The default electrode temperature was set to 43 °C, as recommended by the manufacturer. The electrodes of the SenTec transcutaneous monitor were placed at one of four locations: the left or right upper chest, below the clavicle along the midclavicular line, or the left or right lateral thigh. To enhance measurement validity and safety, electrode sites were assessed routinely (including skin inspection) at scheduled site changes. If local edema, compromised skin integrity, suspected poor perfusion, or inadequate sensor contact was observed, the sensor was repositioned or the measurement site was changed, and pairing was deferred until the displayed TcPCO_2_ value stabilized per the monitor display (no rapid drift over several minutes). After completing the full calibration procedure (10 min), an arterial blood gas sample was obtained and the corresponding TcPCO_2_ value was recorded at that time point rather than a time-averaged value. In addition, we removed the sensor for recalibration and reassessed the patient's skin condition every 2 h of continuous measurement, with four different measurement sites available for alternating use. The calibration was performed according to the standard procedure provided by the SenTec device (12).

The association between PaCO_2_ and TcPCO_2_ was assessed using linear regression (*R*^2^) and repeated-measures correlation (rmcorr, implemented in R V.4.4.0), whereas reliability was evaluated using the intraclass correlation coefficient (ICC). We used repeated measures correlation instead of simple regression or correlation to account for paired measurements taken on multiple occasions for individual patients (13). An rmcorr ≥ 0.75 was considered to indicate a strong within-subject association (trend tracking), whereas an ICC value of 0.6 (moderate reliability) or higher was deemed acceptable. This study considered an ICC value of at least 0.8 to indicate good reliability, as was reported in another study (11).

Agreement between PaCO_2_ and TcPCO_2_ measurements was assessed using the repeated-measures Bland–Altman method. Precision was defined as 1.96 standard deviation of the differences (corresponding to the half-width of the 95% limits of agreement). This study considered an accuracy of less than 5 mmHg, with 95% limits of agreement ranging from ±7.5 to ±11.25 mmHg, to be clinically acceptable (9, 11).

Because multiple PaCO_2_–TcPCO_2_ pairs were available for many infants, we used statistical methods that account for within-subject clustering. Repeated measures correlation (rmcorr) was applied to estimate the association between PaCO_2_ and TcPCO_2_ while controlling for between-subject variability. ICC was calculated using a two-way random-effects model with absolute agreement to assess reliability. For Bland–Altman analyses, we included all paired measurements and estimated the bias and 95% limits of agreement while accounting for multiple observations per individual (13–15).

As a sensitivity analysis to evaluate the impact of unequal numbers of repeated measurements per infant, we repeated key analyses using patient-level averaged PaCO_2_ and TcPCO_2_ values. Specifically, PaCO_2_ and TcPCO_2_ values were averaged within each infant across all available paired measurements, and the association and agreement were re-assessed using correlation (*r*) and Bland–Altman analysis on these infant-level means. This sensitivity analysis was performed to ensure that the results were not driven by infants contributing a large number of paired measurements.

## Results

In this study, 143 neonates with a mean gestational age of 35 ± 3.2 weeks and a mean birth weight of 2,374 ± 694.4 g were recruited. Their perinatal characteristics are listed in Table 1. Among the cohort, there were 88 preterm neonates (gestational age <37 weeks) and 21 VLBW infants (birth weight <1,500 g), as listed in Table 1. These groups were not mutually exclusive. A total of 410 PaCO_2_ and TcPCO_2_ measurement pairs were available for comparison, and the electrode placement was as follows: right upper chest: 265 pairs (65%), left upper chest: 53 pairs (13%), right thigh: 54 pairs (13%), and left thigh: 38 pairs (9%). The median number of paired measurements per infant was 2, with a mean of 3 (range shown in Supplementary Table S2). There were 318 paired PaCO_2_–TcPCO_2_ measurements from preterm neonates, and 142 paired PaCO_2_–TcPCO_2_ measurements from VLBW infants, as listed in Table 2.

**Table 1:** Characteristics of 143 study participants.

Characteristics	Value
Gender, *N* (Male/Female)	92/51
Gestational age at birth, average (mean ± SD), weeks	35 ± 3.2
Birth weight, average (mean ± SD), g	2,374 ± 694.4
TcPCO_2_/PaCO_2_, *n*	410
Gestational age at birth (GA)	
GA ≤ 28 weeks (*N*, %)	8 (6%)
28 weeks < GA ≤ 32 weeks (*N*, %)	17 (12%)
32 weeks < GA ≤ 36 weeks (*N*, %)	63 (44%)
GA ≧ 37 weeks (*N*, %)	55 (38%)
Birth weight (BBW)	
BBW < 1,000 g (*N*, %)	4 (3%)
1,000 ≤ BBW < 1,500 g (*N*, %)	17 (12%)
1,500 ≤ BBW < 2,500 g (*N*, %)	54 (38%)
BBW ≧ 2,500 g (*N*, %)	68 (47%)
Clinical conditions	
Received surfactant treatment	5 (4%)
Hemodynamic significant patent ductus arteriosus (hsPDA)	6 (4.2%)
Required the use of inotropic agents during admission	10 (7%)
Clinical sepsis	37 (26%)

**Table 2:** Subgroup analysis of agreement, association and reliability.

Parameter	Samples	TcPCO_2_/PaCO_2_				
	*n*	Bias/Precision (1.96 SD) (mmHg)	Lower LoA—upper LoA (mmHg)	Linear regression curves, *R*^2^	Repeated measures correlations (rmcorr)	Intraclass correlation coefficient (ICC)
GA < 37 wks	318	+0.96/12.74	−11.8 to +13.7	0.753	0.800	0.93 (95% CI 0.91–0.94, *p* < 0.001)
GA ≧ 37 wks	92	+0.11/11.17	−11.1 to +11.3	0.534	0.730	0.84 (95% CI 0.75–0.89, *p* < 0.001)
GA ≤ 28 wks	96	+0.47/10.58	−10.1 to +11.1	0.667	0.788	0.9 (95% CI 0.85–0.93, *p* < 0.001)
28 wks < GA ≤ 32 wks	60	+0.46/11.17	−10.7 to +11.6	0.684	0.798	0.91 (95% CI 0.84–0.94, *p* < 0.001)
32 wks < GA ≤ 36 wks	162	+1.40/14.50	−13.1 to +15.9	0.787	0.808	0.93 (95% CI 0.91–0.95, *p* < 0.001)
BBW < 1,500 g	142	+0.55/10.98	−10.4 to +11.5	0.683	0.779	0.91 (95% CI 0.87–0.93, *p* < 0.001)
BBW ≧ 1,500 g	268	+0.89/13.33	−12.4 to +14.2	0.751	0.803	0.93 (95% CI 0.91–0.94, *p* < 0.001)
BBW < 1,000 g	52	+0.15/9.02	−8.9 to +9.2	0.707	0.829	0.91 (95% CI 0.85–0.95, *p* < 0.001)
1,000 ≤ BBW < 1,500 g	90	+0.78/11.96	−11.0 to +12.8	0.654	0.762	0.89 (95% CI 0.84–0.93, *p* < 0.001)
1,500 ≤ BBW < 2,500 g	142	+1.94/14.50	−12.6 to +16.4	0.761	0.797	0.93 (95% CI 0.89–0.95, *p* < 0.001)
BBW ≧ 2,500 g	126	−0.30/11.17	−11.5 to +10.9	0.739	0.845	0.92 (95% CI 0.89–0.95, *p* < 0.001)

Bland-Altman analysis presents as bias and 95% LoA (limits of agreement); GA, gestational age; Wks, weeks; BBW, birth weight.

The monitored TcPCO_2_ values ranged from 20.6 to 131 mmHg. We reviewed the clinical records at the time when extreme TcPCO_2_ values were recorded and found no documented evidence of concurrent hemodynamic instability (e.g., hypotension or impaired perfusion) or concurrent sepsis-related deterioration. These values were not excluded and were retained in the statistical analysis. The distribution of the ventilator modes among the total 410 paired measurements was as follows: oxygen flow in 4 (1%) pairs, nasal continuous positive airway pressure in 194 (48%) pairs, noninvasive positive pressure ventilation in 79 (19%) pairs, continuous mandatory ventilation or synchronized intermittent mandatory ventilation in 115 (28%) pairs, and high-frequency oscillatory ventilation in 18 (4%) pairs. The types of ventilation are presented in Supplementary Table S1. Among the preterm neonates, 64.3% of the data were obtained during noninvasive respiratory support, while 35.7% were obtained during invasive mechanical ventilation. Among term neonates, 4.3% of the data were obtained while receiving oxygen therapy alone, 76.1% during noninvasive respiratory support, and 19.6% during invasive mechanical ventilation.

Among these infants, 5 received surfactant treatment for respiratory distress syndrome, and 6 had hemodynamic significant patent ductus arteriosus (hsPDA). Thirty-seven neonates were diagnosed with clinical sepsis; 5 experienced more than one episode. Additionally, 10 infants required the use of inotropic agents during admission (Table 1).

**Figure 2:**
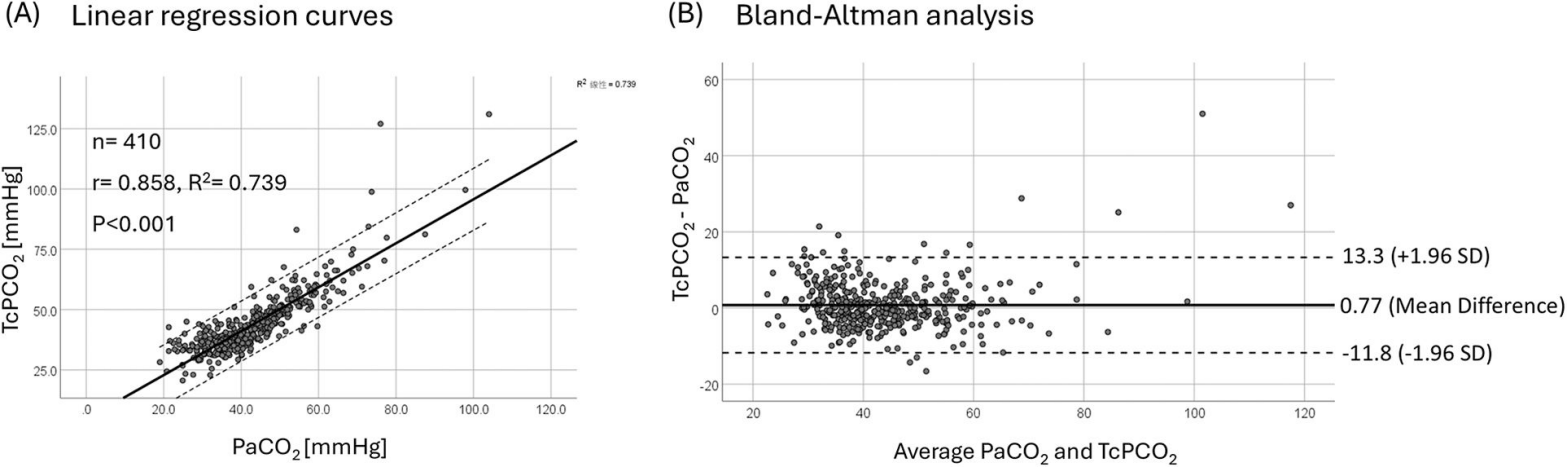
Linear regression curves **(A)** and bland-altman analysis **(B)** of PaCO_2_/TcPCO_2_.

A strong linear correlation was observed between PaCO_2_ and TcPCO_2_ among all paired samples (*R*^2^ = 0.739, *p* < 0.001; Figure 2A). The rmcorr was 0.793, and the ICC for PaCO_2_ and TcPCO_2_ reached 0.92 (95% CI: 0.91–0.94, *p* < 0.001). Bland–Altman analysis revealed a bias of +0.77 mmHg and a precision of 12.5 mmHg (95% LoA: −11.8 to 13.3 mmHg) for TcPCO_2_ relative to PaCO_2_ (Figure 2B).

In the sensitivity analysis using infant-level averaged values (one observation per infant), TcPCO_2_ remained strongly associated with PaCO_2_ (*r* = 0.84, *p* < 0.001). Bland–Altman analysis showed a mean bias of +1.44 mmHg, with a precision of 11.2 mmHg and 95% limits of agreement ranging from −9.7 to +16.8 mmHg (Supplementary Figure S3). This analysis was intended as a robustness check for unequal measurement counts, not as the primary estimate of single-measurement agreement.

### Gestational age (GA)

**Figure 3:**
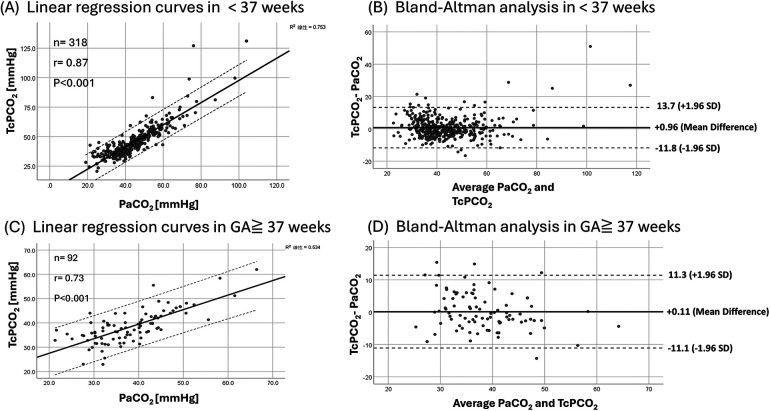
Linear regression curves **(A,C)** and bland-altman analysis **(B,D)** of PaCO_2_/TcPCO_2_ in GA < 37 weeks and GA ≧ 37 weeks.

The gestational age subgroups included GA ≤ 28 weeks (*n* = 8 infants; 96 pairs), 28 < GA ≤ 32 weeks (*n* = 17; 60 pairs), 32 < GA ≤ 36 weeks (*n* = 63; 162 pairs), and GA ≥ 37 weeks (*n* = 55; 92 pairs). TcPCO_2_ demonstrated a strong correlation with PaCO_2_ in preterm infants (GA < 37 weeks), with good reliability (rmcorr = 0.8, ICC = 0.93, 95% CI: 0.91–0.94, *p* < 0.001). Bland–Altman analysis indicated a small positive bias, although the limits of agreement indicated clinically relevant variability (bias +0.96 mmHg, precision 12.74 mmHg, 95% LoA: −11.8 to +13.7 mmHg). In infants with GA ≥ 37 weeks, correlation and reliability were lower than in the preterm group, while bias remained small and limits of agreement were narrower than those observed in the preterm group (Figure 3, Table 2).

### Birth weight (BBW)

**Figure 4:**
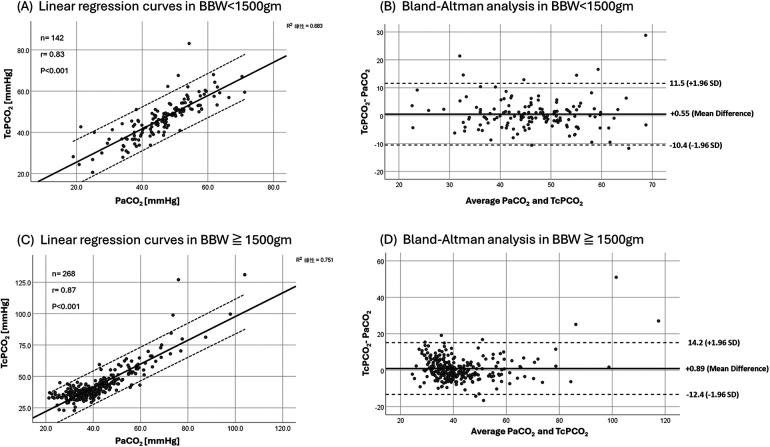
Linear regression curves **(A,C)** and bland-altman analysis **(B,D)** of PaCO_2_/TcPCO_2_ in BBW < 1,500 g and BBW 1,500 g.

Similar findings were observed across birth-weight strata. Birth-weight subgroups included BBW < 1,000 g (*n* = 4; 52 pairs), 1,000 ≤ BBW < 1,500 g (*n* = 17; 90 pairs), 1,500 ≤ BBW < 2,500 g (*n* = 54; 142 pairs), and BBW ≥ 2,500 g (*n* = 68; 126 pairs). In VLBW infants, TcPCO_2_ was strongly correlated with PaCO_2_ and showed good reliability (rmcorr = 0.779; ICC = 0.91, 95% CI 0.87–0.93; *p* < 0.001), with minimal mean bias on Bland–Altman analysis (bias +0.55 mmHg; precision 10.98 mmHg; 95% LoA −10.4 to +11.5 mmHg). In infants with BBW ≥ 1,500 g, correlation and reliability remained high, but the limits of agreement suggested greater variability despite minimal systematic bias (Figure 4, Table 2).

We further stratified gestational age and birth weight (BBW) into more detailed subgroups, and the results are presented in Supplementary Figures S1, S2 and Table 2. These exploratory analyses suggested stronger association/reliability in smaller and more premature infants (e.g., GA < 32 weeks or BW < 1,500 g).

## Discussion

In this study, we found that TcPCO_2_ provided small mean bias and was useful for continuous trend monitoring of PaCO_2_, particularly in very preterm and VLBW infants. These findings suggest that TcPCO_2_ can be used to guide ventilatory management and reduce the frequency of arterial blood sampling in the first weeks of life, when fluctuations in CO_2_ are strongly linked to severe morbidities such as intra-ventricular hemorrhage and bronchopulmonary dysplasia.

Using the prespecified criteria, the mean bias between TcPCO_2_ and PaCO_2_ was small, indicating minimal systematic error. However, the overall 95% limits of agreement (approximately −11.8 to +13.3 mmHg) slightly exceeded the predefined range. This highlights that, while TcPCO_2_ can provide clinically useful continuous trend monitoring in neonates—as supported by prior neonatal studies (9, 11)—the variability in absolute values is not negligible. Accordingly, TcPCO_2_ should be interpreted as a complementary bedside tool rather than a direct substitute for arterial PaCO_2_, particularly in extremely preterm/VLBW infants or when rapid ventilator adjustments are being considered. Confirmatory blood gas sampling remains advisable when TcPCO_2_ values are extreme, when clinical status is unstable, or when tissue perfusion may be compromised.

Our subgroup analyses further illustrate how TcPCO_2_ performance varies across gestational age and birth weight strata. The agreement between TcPCO_2_ and PaCO_2_ was generally better in very preterm and VLBW infants than in term neonates. In this high-risk group, both the correlation coefficients and ICC values were high, and the Bland–Altman analyses demonstrated small biases, while the limits of agreement indicated non-negligible variability, supporting use for trend monitoring rather than interchangeability, which is consistent with the hypothesis proposed by Hochwald et al. Their study suggested that the thinner skin layer in preterm infants may lead to TcPCO_2_ measurements having higher reliability (5). From a clinical perspective, this implies that TcPCO_2_ may be particularly useful as a trend monitoring tool in the smallest infants, while in more mature neonates it should be interpreted with slightly more caution and complemented by periodic arterial blood gases.

The Sentec OxiVent system was evaluated in very preterm neonates (<32 weeks of gestational age), with a TcPCO_2_ bias of +4.7 mmHg and a precision of ±12.3 mmHg reported by Weteringen et al., similar to the findings in this study (10). The study's subgroup analysis indicated that gestational age at birth affected bias because of variations in skin thickness. However, their study used different sensor temperature settings, namely 42 °C for neonates born at ≤25 weeks and 43 °C for those born at 26–31 weeks, which may have affected their statistical interpretation. By contrast, the present study maintained a consistent sensor temperature of 43 °C for all cases and observed improved bias in neonates born at ≤32 weeks.

The overall PaCO_2_–TcPCO_2_ bias and precision (mean ± 1.96 SD) were 0.77 ± 12.5 mmHg, which is consistent with the findings of Tingay et al., who reported a bias of 0.97 mmHg and a precision of 10.4 mmHg in 21 ventilated infants requiring road transport (16). In another study, Tingay et al. observed a bias of 0.8 mmHg and a precision of 13.0 mmHg in 50 ventilated postsurgical neonates (17). The TcPCO_2_ sensor temperature in the present study was set at 43 °C, which is the same as that employed in these previous studies and ensured accurate TcPCO_2_ measurements.

In the overall Bland–Altman analysis, the bias was greater than 0 in nearly all groups (Table 2). This outcome can be attributed to the transcutaneous measurement method, which is affected by skin integrity and local blood perfusion, which may have led to higher TcPCO_2_ values. These findings are in agreement with the assumptions and results of previous studies (5, 9).

Werther et al. reported a low ICC of 0.59 between TcPCO_2_ and PaCO_2_, with a bias of 5.4 mmHg and a precision of 17.2 mmHg (11). Their study included only 25 infants, used a sensor temperature of 41 °C, and incorporated capillary blood samples from the heel or fingertip. Poor circulation at the sensor application site may have affected the TcPCO_2_ accuracy because of the lower sensor temperature. In addition, the authors did not compare all TcPCO_2_ measurements with PaCO_2_ values, which may have affected the overall correlation results (11). By contrast, the present study included only standard compliant PaCO_2_ measurements for paired statistical analysis with TcPCO_2_ to minimize inaccuracies in reliability assessment.

Baumann et al. reported a TcPCO_2_ bias of +4.6 mmHg and a precision of ±13.5 mmHg in neonates born at ≥34 weeks of gestation (9). Sensor temperatures of 42 and 43 °C were used, and similar bias values were obtained at both settings. However, the 43 °C group had a narrower 95% CI for the limits of agreement (9). The bias and precision observed in this study were comparable to those reported for the 43 °C group.

Higher TcPCO_2_ levels are typically detected at high sensor temperatures, likely because of two factors: (a) increased CO_2_ solubility and (b) additional CO_2_ production from skin cell metabolism and tissue diffusion (6, 18). Although high sensor temperatures generally reduce bias, they potentially also increase the risk of thermal injury, particularly in preterm neonates (18). However, no instances of skin injury were observed in the current study, even among preterm infants. Based on these findings, a sensor temperature of 43 °C appears to be both safe and effective for TcPCO_2_ monitoring in preterm neonates.

The reported bias was very small (generally clustered around 0.67 ± 0.63 mmHg); therefore, any inaccuracy in instrument calibration could significantly affect the study results. To minimize such effects, all operators received standardized training before data collection, and a rigorous SOP for electrode calibration was implemented to ensure consistency and reduce bias arising from the calibration procedure itself.

In the grouped statistical analysis of GA ≤ 28 weeks, 28 weeks < GA ≤ 32 weeks, and 32 weeks < GA ≤ 36 weeks, the strongest correlation was observed in the 32 weeks < GA ≤ 36 weeks group. Although the bias and precision were more favorable in the GA ≤ 28 weeks group, possibly because extremely preterm infants have thinner and less developed skin, which may have minimized interference with TcPCO_2_ monitoring. However, only eight patients were enrolled in the GA ≤ 28 weeks group, which is likely insufficient to provide confidence in these subgroup results. Given the small sample size in some strata (e.g., GA ≤ 28 weeks and BBW < 1,000 g), these subgroup findings should be interpreted as exploratory and hypothesis-generating rather than definitive.

In a comparison of birth weight groups, Werther et al. reported an *r* value of 0.6, a bias of +5.4 mmHg, and a precision of ±17.2 mmHg with an ICC of 0.59 between PaCO_2_ and TcPCO_2_ in a cohort of 25 infants (11). They further categorized neonates into BBW < 1,500 g and BBW ≥ 1,500 g groups but did not observe improved correlation or accuracy. This may be because they included capillary blood samples from the heel or fingertip in their PaCO_2_ data. Furthermore, TcPCO_2_ monitoring in their study was conducted using the SenTec V-Sign Sensor set at 41 °C (11). By contrast, the current study revealed strong correlation and accuracy, even in the subgroup analysis of BBW < 1,500 g and BBW < 2,500 g groups. These findings support the reliability of TcPCO_2_ monitoring for continuous assessment in neonates with both low birth weights and VLBWs. Our findings are consistent with Borenstein-Levin et al. (19), who reported small mean bias but wide limits of agreement in ELBW infants, supporting TcPCO_2_ as a complementary tool for trend monitoring rather than a direct substitute for blood gas measurements.

Although transcutaneous CO_2_ monitors are valuable for tracking ventilation status, they should not be solely relied upon for making significant patient care decisions. Additional confirmation, ideally through arterial blood gas analysis, should be conducted because extreme values may occur in neonates with highly unstable vital signs. Baumann et al. noted that in patients at risk for hemodynamic instability, compromised peripheral perfusion may contribute to wide precision intervals (9). In addition, Hochwald et al. reported that skin edema, poor tissue perfusion, and acidosis can affect the correlation between TcCO_2_ and PaCO_2_ (5). They also reported that technical limitations may affect TcCO_2_ reliability, with such limitations involving, for example, problems with sensor preparation, problems with sensor positioning, high humidity in incubators, and the need for frequent sensor site changes to prevent skin trauma (5). Among our enrolled patients, some did have hypotensive and/or on inotropes, and each record included blood pressure, heart rate, body temperature, hemoglobin, and ventilator settings. However, no abnormal extreme values were observed in these patients; therefore, these cases were not specifically analyzed separately. Also, we still included paired data with markedly discrepant values; however, such data points became outliers in the analysis. Although a difference of ±7.5 to ±11.25 mmHg is considered clinically acceptable in routine practice, both hypercarbia and hypocarbia are associated with respiratory and neurologic complications, particularly in preterm infants. Also extreme values may be more likely to occur in patients with clinically unstable conditions. Therefore, we consider that TcPCO_2_ values exceeding 60 mmHg or below 30 mmHg should be confirmed by arterial blood gas analysis (5).

Overall, TcPCO_2_ accuracy is influenced by skin integrity and local tissue perfusion. Conditions such as hypotension, inotropic/vasoactive support, sepsis-related circulatory changes, edema, and hsPDA may reduce peripheral perfusion and increase variability between TcPCO_2_ and PaCO_2_, potentially widening the limits of agreement. Ventilator mode may also reflect disease severity and clinical sampling frequency, which can indirectly affect observed agreement in retrospective datasets. Although we reported the prevalence of these clinical conditions, the retrospective design and the relatively small number of unstable infants limited our ability to adjust for time-varying perfusion status or to conduct adequately powered stratified analyses for all factors. Clinically, TcPCO_2_ should therefore be interpreted primarily as a trend-monitoring tool; in unstable infants or when tissue perfusion is suspected to be compromised, arterial blood gas confirmation is recommended, particularly for extreme TcPCO_2_ values or when major ventilator changes are being considered.

## Limitations

The present study has several limitations, including sample cohort distribution, timing of sampling, multiple sampling per patient, and different medical conditions of patients. The timing of blood sampling was determined by each patient's clinical status and clinical needs, making it impossible to obtain TcPCO_2_ and PaCO_2_ measurements at standardized or pre-specified intervals. As a result, the number of paired TcPCO_2_/PaCO_2_ measurements varied substantially among patients, and uniform measurement frequency could not be achieved across the cohort. Also, because of the limited sample size, confounding variables such as hypotension and sepsis, which can affect tissue perfusion and TcPCO_2_ measurements, were not controlled for. Although we reported the prevalence of clinical sepsis and inotropic support in the cohort, we lacked sufficiently detailed time-varying information to adequately adjust for these factors or perform robust stratified analyses. In addition, the small numbers of infants receiving surfactant (*n* = 5) and requiring treatment for hsPDA (*n* = 6) limited the generalizability of our findings to these specific clinical contexts. These factors may introduce heterogeneity in data collection and could potentially influence the agreement analysis.

Finally, this study was not originally designed exclusively for very preterm or VLBW infants; rather, it included a heterogeneous NICU population. Although we prespecified very preterm and VLBW infants as the primary population of interest for this analysis, residual confounding by clinical condition and treatment strategies across gestational age groups cannot be fully excluded.

## Conclusions

TcPCO_2_ measurement is a noninvasive and accurate method for continuous monitoring of PaCO_2_ trends in neonates, especially in very preterm and/or very low birth weight infants. However, TcPCO_2_ should be interpreted primarily as a trend-monitoring tool; in unstable infants or when tissue perfusion is suspected to be compromised, arterial blood gas confirmation is recommended, particularly for extreme TcPCO_2_ values or when major ventilator changes are being considered.

## Data Availability

The raw data supporting the conclusions of this article will be made available by the authors, without undue reservation.
