# Supplementary figures and images for: Accuracy of noninvasive transcutaneous carbon dioxide monitoring in preterm neonates and very low birth weight infants compared with larger neonates
Source: Front Pediatr. 2026 Mar 31;14:1794358. doi: 10.3389/fped.2026.1794358 (PMC13076325; doi:10.3389/fped.2026.1794358)

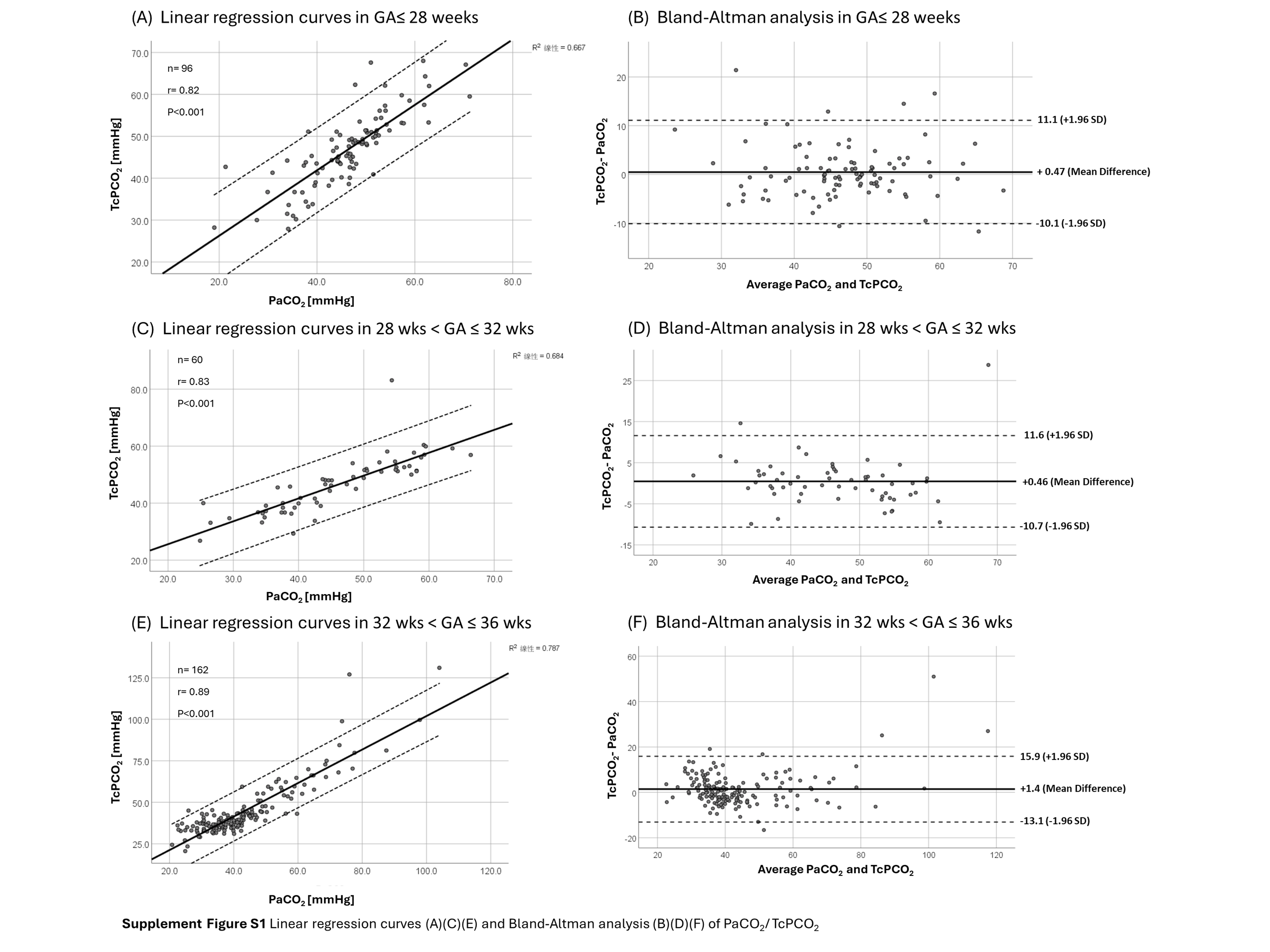

Supplement: Supplementary file 1 [file Image1.tif]

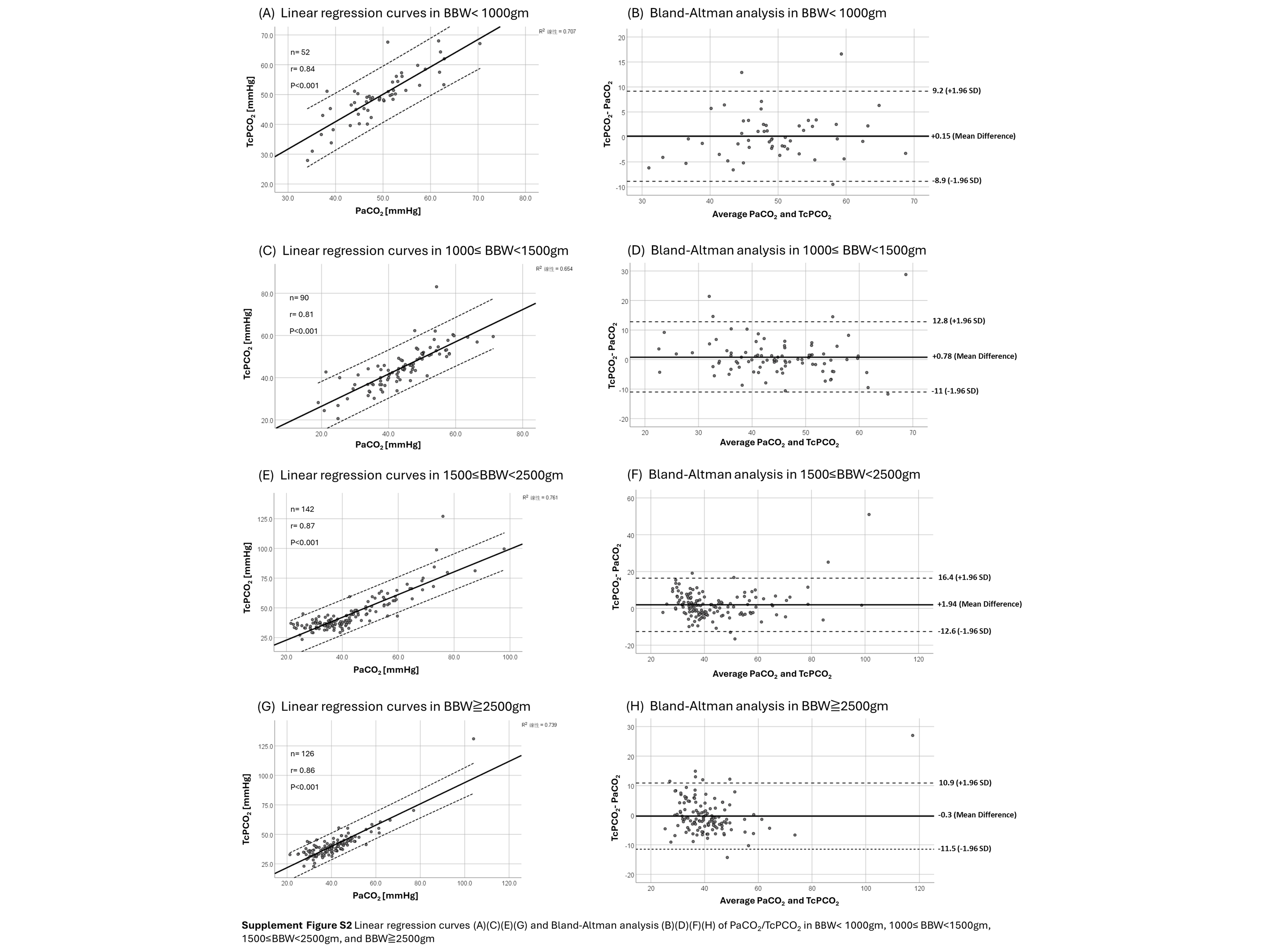

Supplement: Supplementary file 2 [file Image2.tif]

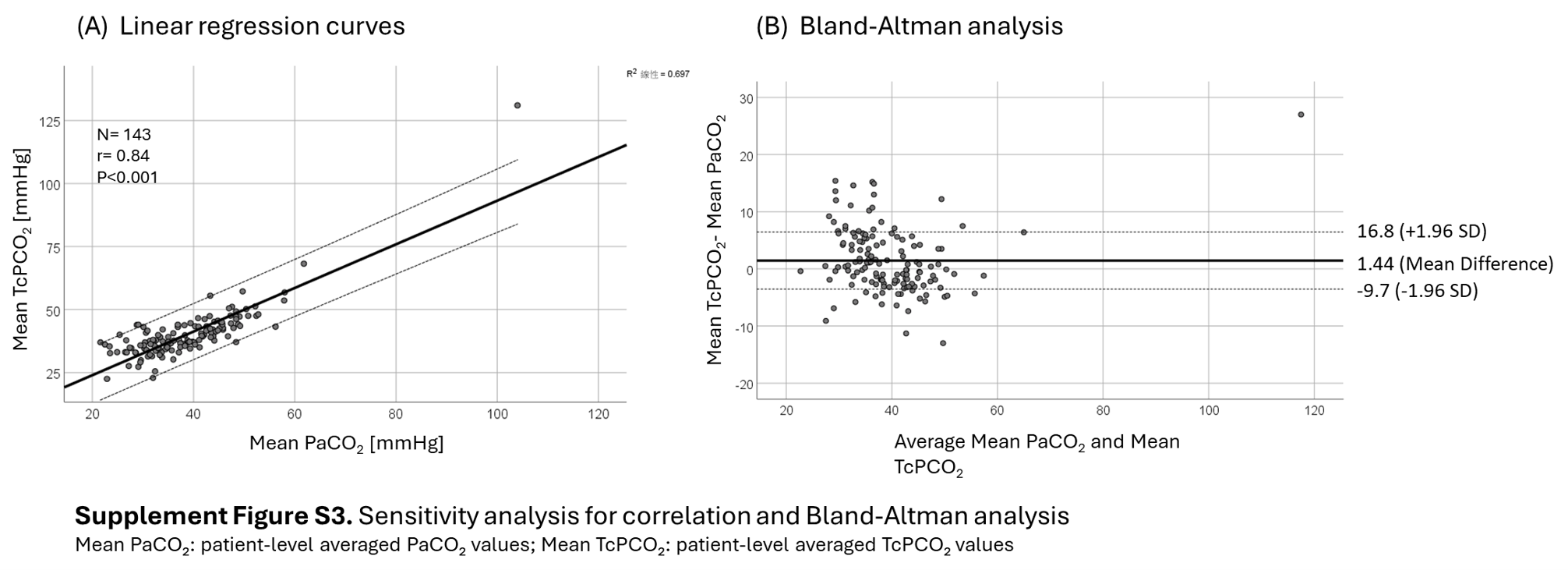

Supplement: Supplementary file 3 [file Image3.tif]

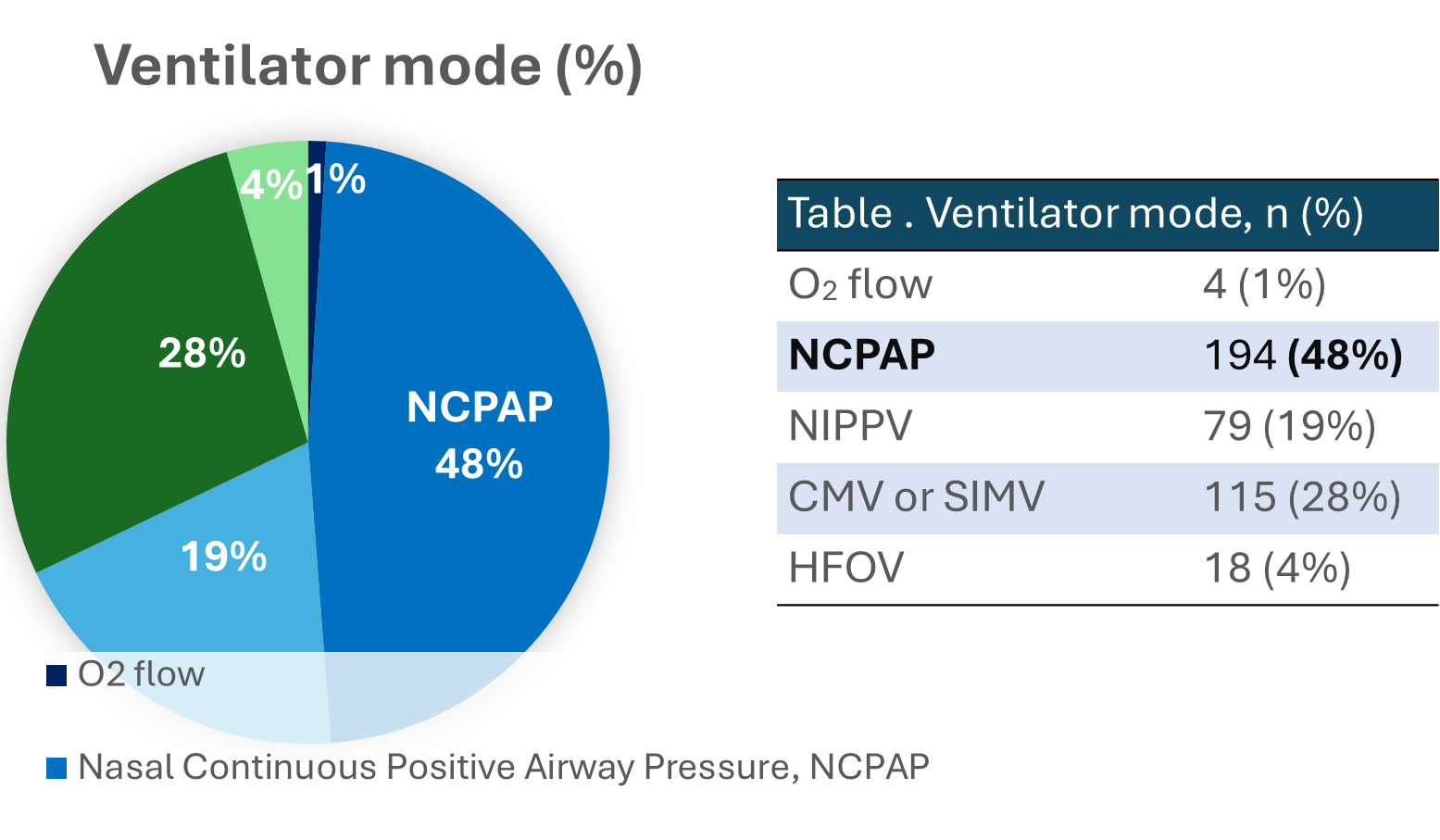


Supplement table S1. Ventilator mode

Supplement: Supplementary file 4 [file Table1.docx]
