# Supplementary material for: Accuracy of noninvasive transcutaneous carbon dioxide monitoring in preterm neonates and very low birth weight infants compared with larger neonates
Source: Front Pediatr. 2026 Mar 31;14:1794358. doi: 10.3389/fped.2026.1794358 (PMC13076325; doi:10.3389/fped.2026.1794358)
Supplement: Supplementary file 5 [file Table2.docx]

Supplement Table S2. Number of paired measurements per infant

| Median | Average | Range | | | |
| --- | --- | --- | --- | --- | --- |
|  |  | <10 | 10~20 | 20~30 | 30~40 |
| 2 | 3 | 134 | 6 | 2 | 1 |
